# Low-Field Magnetic Resonance Imaging Findings in 18 Dogs With Presumed Optic Neuritis

**DOI:** 10.3389/fvets.2020.585828

**Published:** 2021-01-07

**Authors:** Laura Muñiz Moris, Giunio Bruto Cherubini, Abby Caine

**Affiliations:** Dick White Referrals, Cambridgeshire, United Kingdom

**Keywords:** canine optic nerve, granulomatous meningoencephalitis, meningoencephalomyelitis of unknown etiology, magnetic resonance imaging, optic neuritis

## Abstract

Canine optic neuritis has been attributed to a focal or disseminated form of granulomatous meningoencephalitis (GME) amongst other etiologies. Magnetic resonance imaging (MRI) has been proven to help differentiate the structures within the optic nerve sheath and therefore could aid the diagnosis of optic neuritis in dogs. The objectives of this study were to describe and compare the MRI abnormalities affecting the optic nerve sheath complex and optic chiasm in dogs with clinically suspected optic neuritis as a component of meningoencephalitis of unknown etiology (MUE) or as an isolated form (I-ON). Retrospective evaluation of patient details, clinical signs, cerebrospinal fluid (CSF) analysis, and MRI findings of dogs with clinically suspected optic neuritis between January 2011 and May 2018 was performed. Eighteen dogs met the inclusion criteria. MRI findings included contrast enhancement of both optic nerves (11/18) and optic chiasm (6/18), changes within the CSF volume surrounding the optic nerve (10/18), changes to the optic disc (10/18), changes of size or signal affecting the optic chiasm (10/18), changes in the Short TI inversion recovery (STIR) signal of the optic nerve (7/15), retrobulbar changes (3/18), and concurrent brain lesions (13/18). A variety of subtle MRI features may indicate optic nerve involvement and low-field MRI is a sensitive method to detect changes within the optic nerve sheath complex in dogs with optic neuritis as an isolated form (I-ON) or as an extension of MUE.

## Introduction

Optic neuritis (ON) is an inflammation of the optic nerve (1) and in dogs presents as a clinical syndrome characterized by unilateral or bilateral acute vision loss and altered pupillary light reflexes (2). Optic neuritis has recently been classified in multifocal meningoencephalitis of unknown etiology (MUE-associated ON), isolated optic neuritis (I-ON) and “others” (2). Previously described etiologies of canine optic neuritis include infectious diseases, inflammatory central nervous system (CNS) diseases [such as granulomatous meningoencephalitis (GME)], trauma, orbital diseases, neoplastic processes, toxins, vitamin A deficiency, and idiopathic (1). In a previous review of GME cases (3), 15 out of 151 dogs with GME showed concurrent signs of optic neuritis. GME is a histopathological diagnosis of a subtype of MUE (4, 5) and can develop in a focal, disseminated or ocular form; however, both the ocular and disseminated forms can have concurrent CNS and optic nerve lesions (6).

In this study, the authors will focus on the inflammatory CNS diseases causing optic neuritis and will use the term MUE (isolated or disseminated) for all the cases since no histopathological analysis was available.

The optic nerve is a CNS tract that is surrounded by cerebrospinal fluid (CSF), which is in continuation with the subarachnoid space and contained within a sheath that is an extension of the meninges (7). Differentiation between the optic nerve and surrounding CSF, margin of the optic nerve sheath, surrounding intraconal fat and extraocular muscles in healthy dogs has been described using a low field MRI in a previous study (8).

There are previous publications describing some MRI features of MUE-associated canine optic neuritis. One study (9) described optic nerve and optic chiasmal hyperintensities, along with hyperintense patchy brain lesions and contrast enhancement of the optic nerve, another study (10), described one case of T2W and STIR hyperintense and contrast enhancing optic nerves, and one case report (11) of a dog with presumed ocular GME described contrast enhancing optic nerves with a concurrent swollen contrast enhancing optic chiasm in addition to T2W hyperintense lesions in the thalamus and areas of contrast enhancement affecting the cervical spinal cord. There is however a lack of publications describing the MRI findings in this condition in a larger cohort of dogs and to the authors' knowledge, abnormalities affecting the different structures contained within the optic nerve sheath complex in dogs with optic neuritis have not been previously described.

The objective of this study was to describe the presence and frequency of subtle MRI abnormalities affecting the optic nerve sheath complex and optic chiasm in dogs with clinical symptoms suggesting optic neuritis as a component of MUE or as an isolated form.

We hypothesized that patients with optic neuritis as a component of MUE will have multiple MRI visible abnormalities of the optic nerves related to the optic nerve sheath complex.

## Materials and Methods

This was a retrospective descriptive study. Medical records, between January 2011 and May 2018, were searched for dogs that presented to a referral veterinary hospital with a clinical suspicion of optic nerve pathology.

Dogs were included in this study if (a) they had central visual deficits, assessed by an absent or reduced menace response (search terms included: blind, blindness, vision, visual and menace), (b) underwent an MRI examination with at least one sequence provided to specifically evaluate the optic nerves, and (c) had appropriate clinical signs and met one or more of the following criteria for the diagnosis of MUE: (i) MRI findings consistent with MUE (12), (ii) CSF sample supporting inflammatory disease, and/or (iii) positive response to immunomodulatory treatment. These multiple criteria were used to diagnose MUE in the absence of a single ante mortem diagnostic test.

Dogs were excluded from the study if they showed decreased or absent vision due to primary intraocular or retrobulbar pathology (such as retrobulbar masses or retrobulbar abscesses), or if the MRI studies were considered of insufficient image quality for retrospective review.

For all cases, patient details, clinical signs, cerebrospinal fluid (CSF) analysis, MRI findings and treatment response were retrospectively reviewed and recorded. Infectious diseases (Toxoplasma and Neospora) were excluded based on serology tests and/or CSF analysis.

The CSF analysis result was considered suggestive of inflammatory disease when the results showed increased nucleated cell count (>6 cells/μL), mononuclear or mixed pleocytosis and/or elevation in proteins (>0.30 g/l) (13).

Three cases without CSF were included in the study based on the clinical examination, MRI findings and positive response to immunomodulatory treatment.

MRI images were acquired using a 0.4 tesla MRI scanner (Aperto Lucent, Hitachi Medical Corporation, Tokyo, Japan). All patients were scanned under general anesthesia; different anesthetic protocols were used for premedication and induction depending on the assessment of the attending anesthetist. The anesthesia was maintained with isoflorane (Isothesia 100 mg/g, Henry Schein, New york, USA) and oxygen. The dogs were positioned in sternal recumbency with the head positioned inside a human knee coil.

A complete MRI brain protocol included the following sequences: T2W sagittal, T2W, Fluid-Attenuated Inversion Recovery (FLAIR), T2^*^W gradient-echo and T1W transverse. This protocol was performed in all cases except in three, where the FLAIR sequence was not available.

Additional sequences for evaluation of the optic nerves were: Short TI inversion recovery (STIR) dorsal oblique aligned with the orbit (performed in thirteen cases), T1W dorsal oblique (performed in ten cases) and sagittal oblique aligned with the optic nerve (performed in three cases) and 3DT1 dorsal Fat Saturated (FS) oblique aligned with the optic nerve (performed in three cases). The T1 Weighted (T1W) sequences were acquired pre- and post- paramagnetic contrast administration (Gadovist 1.0 mmol/ml, Bayer, Healthcare, Leverkusen, Germany) at 0.1 mmol/kg of body weight. The sequences to evaluate the optic nerves were acquired depending of the preference of the radiologist at the time of the MRI. Measurement of the optic nerves was attempted however was not considered reproducible and therefore measurements were not included in this study. The technical parameters for the MRI sequences used in this study are represented on Table 1.

**Table 1:** Representing the technical parameters for the MRI scans used in this study.

**MRI complete brain protocol**	**Slice thickness (mm)**	**TR**	**TE**
•T2W spin-echo sagittal and transverse	3 and 3-4	5342 and 6613	126
•FLAIR transverse	3-4	9518	90
•T2W gradient-echo transverse	3-4	820	40
•T1 spin-echo transverse pre and post contrast	3-4	529	12.9
**Optic nerve sequences**	**Slice thickness (mm)**	**TR**	**TE**
•STIR dorsal	3-4	4611	60
•T1 spin-echo dorsal and sagittal oblique	3-4	380	15
•3DT1 gradient-echo dorsal oblique FS	1	50.4	6.7

*MRI, magnetic resonance imaging; TR, repetition time; TE, echo time*.

The MRI studies were reviewed retrospectively by a board certified radiologist and an imaging intern collaboratively. The reviewers were blinded to the MRI results but not to the final diagnosis. The assessed MRI features are detailed in Table 2.

**Table 2:** Representing the evaluated structures, the observed MRI features and the sequences evaluated for each feature.

**Structures evaluated**	**Features identified**	**Sequences and planes evaluated**
•Optic nerve sheaths	•Absence/Presence of CSF	•T2 Transverse, T2 Sagittal
•Optic discs	•Swollen if sessile bulge into the vitreous chamber	•T2 sagittal, STIR dorsal oblique
•Optic nerves	•Hyperintense/normal signal intensity	•STIR dorsal oblique
•Optic chiasm	•Enlarged if in contact with the thalamic hemispheres or interthalamic adhesion	•T2 sagittal, T2 transverse
•Retrobulbar space	•Hyperintensity and/or contrast enhancement beyond normally expected (8), or contrast enhancement, of the retrobulbar muscles and fat	•STIR dorsal oblique, T1 pre and post contrast dorsal oblique
•Optic nerve and optic chiasm	•Abnormal contrast enhancement unilateral or bilateral	•T1 pre and post contrast transverse, sagittal, and/or dorsal oblique
•Brain lesions	•Any lesion compatible with MUE (12)	•T2 sagittal, transverse, FLAIR transverse, T1 pre, and post contrast transverse

*STIR, Short-TI Inversion Recovery; FLAIR, Fluid-Attenuated Inversion Recovery; MUE, meningoencephalitis of unknown origin; CSF, cerebrospinal fluid*.

## Results

Eighteen dogs met the inclusion criteria (Table 3). Sixteen dogs were diagnosed with suspected optic neuritis as an extension of generalized MUE and two dogs were diagnosed with suspected isolated optic neuritis (I-ON) (cases number 6 and 18).

**Table 3:** Summary of the signalment, MRI findings, CSF results, and outcomes of dogs diagnosed with optic neuritis as an isolated form or secondary to presumed meningoencephalitis of unknown etiology.

**Number**	**Breed**	**Weight (kg)**	**Age (years)**	**Sex**	**Changes STIR**	**Absent CSF**	**Swollen Optic chiasm**	**Contrast enhancement OC**	**Contrast enhancement ONs**	**Orbital changes**	**Optic disc bulging**	**Brain affection**	**CSF**	**Signs of multifocal brain disease**
1	Chihuahua	2.8	5.3	ME	N/A	Y	N	N	N	N	N	Y	N/A	+
2	Chihuahua	3.3	3.4	MN	Y	Y	N	N	Y	N	N	Y	N/A	+
3	Cavalier King Charles Spaniel	6	0.35	FE	Y	N	N	N	N	N	Y (U)	N	N/A	+
4	Cross Breed	6.5	4	FE	N	N	Y	N	Y	Y	N	N	+	+
5	Jack Russell Terrier	7.7	7.25	FN	Y	Y	N	N	Y	N	N	Y	+	+
6	Cross Breed	8.4	9.6	MN	N/A	N	Y	Y	Y	N	N	N	-	-
7	French Bulldog	8.6	1.25	ME	N	Y	Y	N	Y	N	Y (U)	Y	-	-
8	West Highland White Terrier	9.8	7	FS	N	Y	N	N	Y	N	Y (B)	Y	+	+
9	French Bulldog	10.2	0.75	MN	N/A	Y	N	N	Y	N	Y (B)	Y	-	+
10	French Bulldog	10.2	1.25	MN	N	N	Y	Y	Y	N	Y (U)	Y	-	+
11	French Bulldog Cross	10.2	3.4	MN	Y	Y	N	N	Y	Y	Y (U)	Y	-	-
12	Irish Terrier	10.6	7	MN	Y	Y	Y	N	N	N	Y (B)	Y	-	+
13	French Bulldog	10.6	6	FE	N	N	Y	Y	Y	N	N	Y	+	+
14	Lhasa Apso	12.1	6.3	ME	Y	Y	Y	N	N	Y	Y (B)	Y	+	+
15	Irish Setter	16.8	2	FE	N	N	N	N	N	N	N	N	-	-
16	Border Collie	23	7	MN	N	N	Y	Y	N	N	Y (B)	Y	+	+
17	Greyhound	28.4	9.4	ME	N	N	Y	Y	N	N	Y (B)	Y	+	+
18	Golden Retriever	32.4	3.5	FE	Y	Y	Y	Y	Y	N	N	N	-	+

*ME, male entire; MN, male neutered; FE, female entire; FN, female neutered; Y, yes; N, no; B, bilateral; U, unilateral; N/A, not available; +, positive; -, negative*.

### Signalment

The breeds included four large breeds, six medium breeds, and eight small breeds. These included seven neutered males, five entire females, four entire males, and two neutered females. The age of the dogs varied between 9 months and 9 years (mean 4.5 years) and the weight varied between 2.6 and 32.4 kg (mean 12 kg).

### CSF Results

Of the included cases, 15/18 had a CSF sample taken and 7/15 cases had a result supportive of inflammatory CNS disease. The remaining 8/15 CSF results were negative, of which two cases had been treated with glucocorticoids before the sample was obtained. For the three cases included without CSF, these cases did not have a CSF sample taken due to the opinion of the attending neurologist: in two cases it was considered contraindicated due to a suspicion of raised intracranial pressure and the other case had previously been diagnosed with MUE 2 years before, at which point the dog had signs of CNS neurolocalisation without any visual deficits, and had a CSF sample result suggestive of inflammatory CNS disease.

### MRI Findings

**Figure 1:**
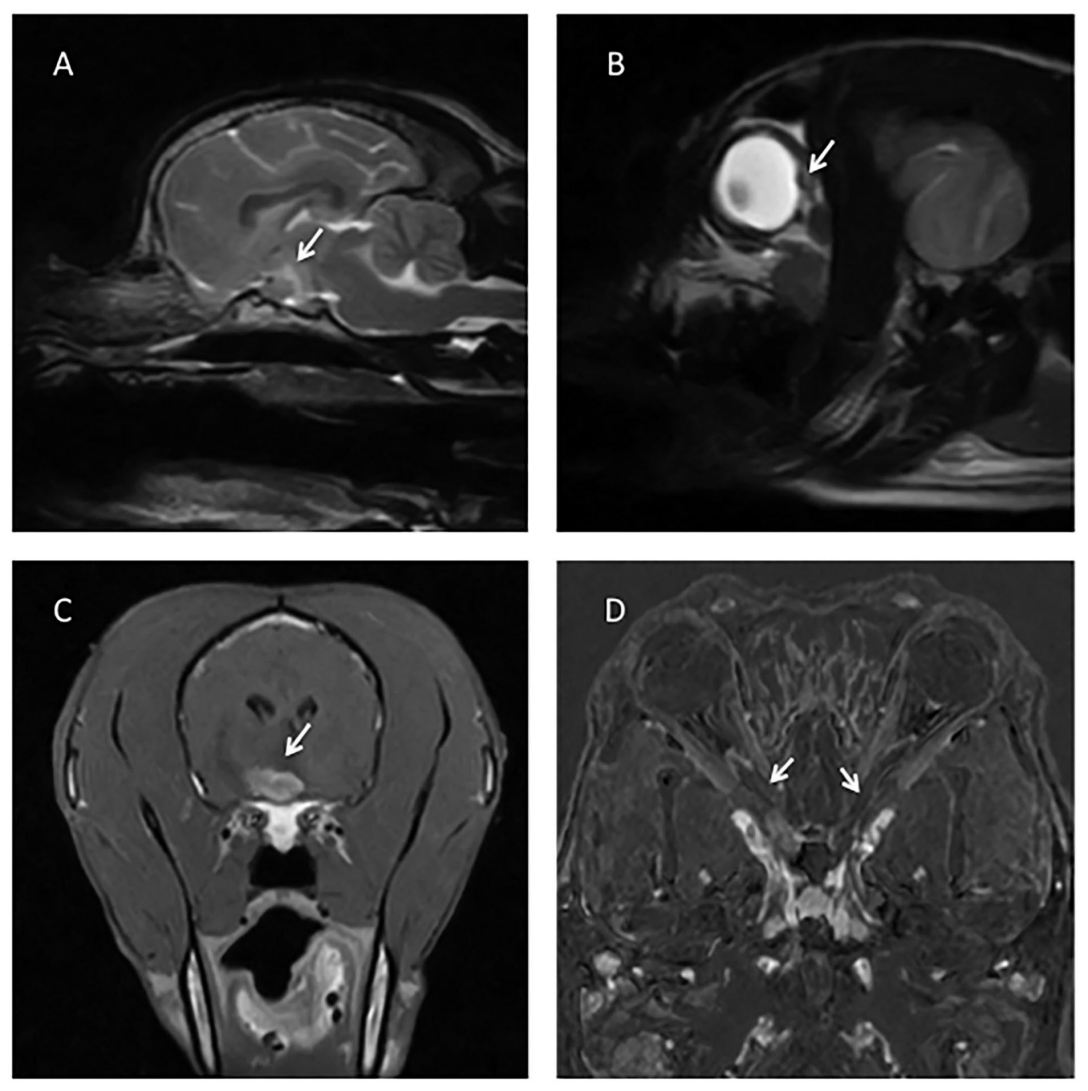
**(A)** T2W sagittal image of the brain showing a swollen optic chiasm (arrow). **(B)** T2W sagittal of the head, showing a bulging optic disc (arrow). **(C)** T1W post contrast transverse of the brain showing a strongly and slightly heterogeneous contrast enhancing of the optic chiasm (arrow). **(D)** Subtraction technique (digitally post-processed subtraction of the pre contrast T1W dorsal oblique sequence from the identical sequence obtained after gadolinium contrast administration), with arrows showing contrast enhancement of both optic nerves.

**Figure 2:**
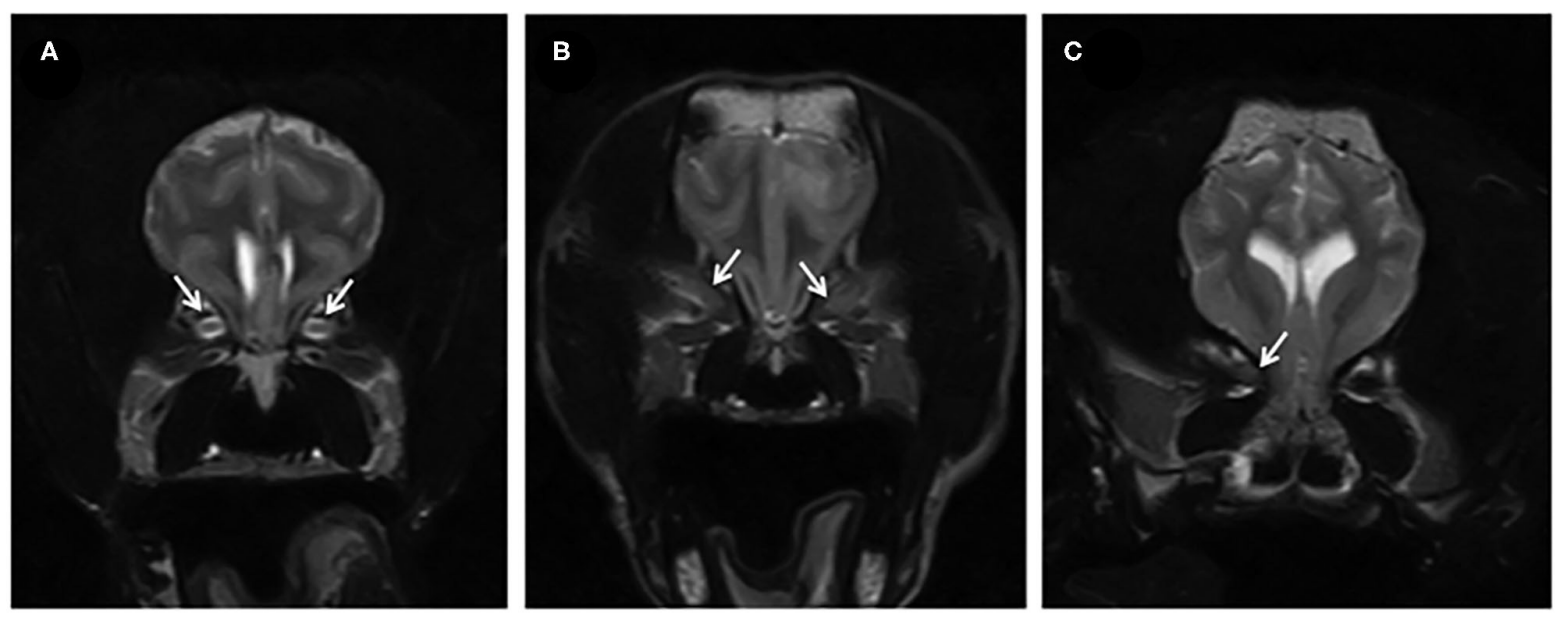
**(A)** T2W transverse of the brain of a dog showing a normal optic nerve sheath complex (arrows). **(B,C)** T2W transverse in two affected dogs, where there is absent CSF surrounding the optic nerve and generalized lack of differentiation of the structures within the optic nerve sheath complex (indicated by the arrows) suggesting swelling of the optic nerves.

**Figure 3:**
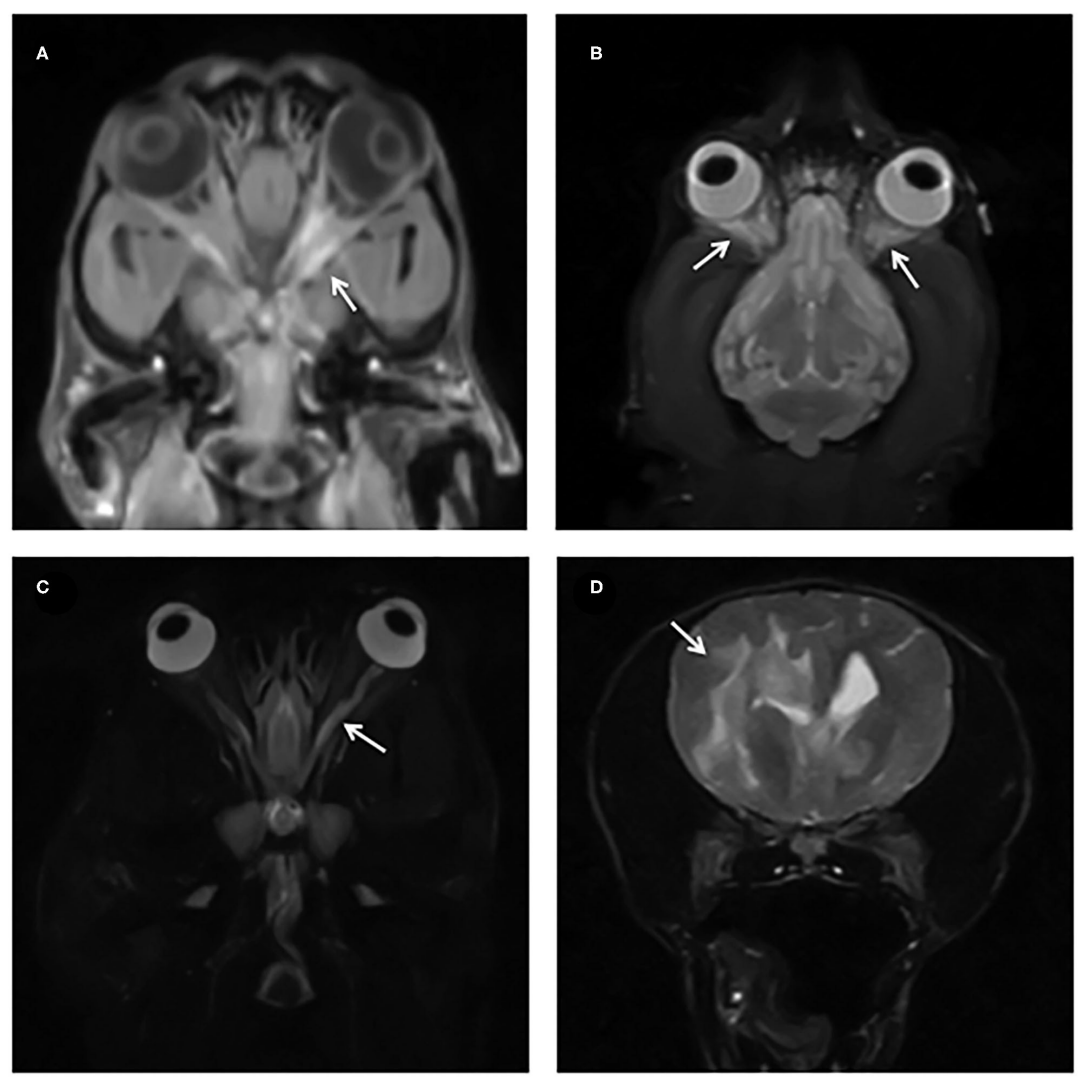
**(A,B)** Dorsal T1W FS post contrast and STIR with arrows showing diffuse contrast enhancement and STIR hyperintensity of the retrobulbar structures. **(C)** STIR dorsal showing a hyperintense optic nerve (arrow). **(D)** T2W transverse images showing extensive T2W hyperintensity tracking along the white matter in a dog with MUE.

The MRI findings affecting the optic nerves and optic chiasm of both the I-ON and MUE-associated ON cases included:

- Optic nerve enhancement (11/18, 61%) (Figure 1D)
- Absent CSF within the optic nerve sheath (10/18, 55%) (Figures 2A–C)
- Optic chiasm swelling (10/18, 55%) (Figure 1A)
- Optic disc swelling (10/18 55%) (Figure 1B), which was bilateral in six cases (60%) and unilateral in four cases (40%)
- Abnormal STIR signal of the optic nerve (7/15, 46%) (Figure 3C)
- Optic chiasm enhancement (6/18, 31%) (Figure 1C)
- Changes to the retrobulbar space (3/18, 16%) (Figures 3A,B)

Regarding the distribution of contrast enhancement, 22% (4/18) had enhancement of both optic chiasm and one or both optic nerves, 38% (7/18) had enhancement of the optic nerve(s) without chiasmal enhancement, 11% (2/18) had enhancement of the optic chiasm alone and 33% (6/18) had neither.

Thirteen cases (13/18, 72%) also had MRI abnormalities of the brain parenchyma consistent with MUE, predominantly multifocal T2W and FLAIR hyperintense patchy lesions affecting mainly the white matter (Figure 3D).

## Discussion

In our study all the cases had subtle abnormalities affecting or surrounding the optic nerve and/or optic chiasm and the majority of the cases (13/18, 72%) showed concurrent MRI changes of the brain.

Contrast enhancement of the optic nerves (observed on T1W sequences on either transverse, dorsal and/or sagittal oblique planes) was the most common finding in our study, present in 61% (11/18) of the cases, and contrast enhancement of the optic chiasm was seen in 31%, which is similarly described in previous reports of optic neuritis (9–11).

A swollen optic chiasm was seen in 55% (10/18) of the cases. A swollen optic chiasm has been described with optic GME in one case report (11) without concurrent swelling of the optic nerves. In that case, histopathology revealed infiltration with lymphocytes, plasma cells, and histiocyte-like epithelioid cells in the dilated microvessels within the optic chiasm, a focal cerebral lesion and one of the optic nerves, which confirmed the diagnosis of GME.

Absent CSF signal within the optic nerve sheath complex has been previously used as a feature for detection of optic neuritis in a recent study (14) but has not been well described in the veterinary literature. This MRI feature was present in 55% (10/18) of the cases. We hypothesized that this could be related to swelling of the optic nerve and/or thickening of the optic nerve sheath. This would cause a reduction of the subarachnoid space surrounding the optic nerves and therefore the amount of CSF would be reduced, resulting in loss of differentiation of the normal optic nerve sheath complex structures. It would be useful to confirm if swelling was present using measurements, but breed and weight based ranges for normal optic nerve sizes are not available. The mean diameter of the optic nerve sheath complex reported in a previous study (8) was 3.7 mm and of the optic nerve 1.7 mm, and these dogs had a mean weight of 17 kg bodyweight. In a second study (15), the mean optic nerve sheath diameter was 3 mm in a population of 100 dogs with a mean of 23.1 kg, of which 22 dogs had suspected increased intracranial pressure. A bodyweight to optic nerve sheath diameter positive correlation has been noted in previous studies in dogs (15, 16). Considering the small variation of the thickness of both the optic nerve and optic nerve sheath complex observed in previous studies, further studies are needed to determine a normal size of the optic nerve in smaller breeds which are over represented with cases of MUE and allowing slight changes in thickness of these structures to be appreciated in clinically relevant cases. Additionally analyzing if absent optic sheath CSF is noted in any cases without MUE/visual deficits could be helpful to help interpret these results further.

Optic disc swelling was present in 55% (10/18) of the cases (six cases were bilateral and four cases were unilateral). Optic disc swelling has been described with papilloedema, optic nerve inflammation (papillitis) and optic nerve neoplasia, such as glioma or meningioma (17). Papilloedema is almost always bilateral and is not associated with visual deficits initially, but can lead to vision loss in later stages. It occurs as a result of increased CSF volume in the optic nerve sheath due to increased intracranial pressure resulting in compression of the optic nerve and swelling of the axons and in dogs this is associated with brain tumors (18). Papillitis was reported in nine of thirteen (69%) cases with optic neuritis by funduscopic examination (9), and a case report of a dog diagnosed with canine distemper virus and secondary optic neuritis showed swollen optic nerves protruding into the posterior segment of the eye (19). Our diagnosis was made based on the MRI changes, and considering that the amount of CSF surrounding the optic nerve in our cases was either absent or normal, these findings are considered more likely to be secondary to optic neuritis related papillitis rather than papilledema.

Another common finding in our study was asymmetry of the STIR signal of the optic nerves. This was present in 46% (7/15) of the cases. STIR sequences were useful to evaluate the optic nerve as it suppressed the fat from the retrobulbar space, providing a T2W type of image highlighting pathology (20). Pathological inflammatory changes within the optic nerve sheath complex (affecting either the nerve or the surrounding sheath) could result in hyperintensity on STIR sequences.

Retrobulbar changes were detected in a low number (16%, 3/18) of dogs. These were associated with ipsilaterally affected optic nerves in MRI and were presumed to be due to an extension of the underlying inflammatory process. The presence of retrobulbar changes could therefore be secondary to optic nerve pathology and a primary cellulitis of the retrobulbar space should not be assumed.

Seventy-two percent (13/18) of the cases had concurrent brain lesions, indicating a disseminated form of MUE that was affecting also the optic nerves. Twenty-eight percent (5/18) of the cases did not have any brain abnormalities, and therefore could be considered as I-ON according to MRI findings alone. However, the majority of these cases (3/5) presented neurological signs suggesting multifocal brain disease as well as visual deficits, demonstrating a more disseminated form of MUE without identified MRI changes of the brain. This has been previously described in other studies (21–23). The remaining two cases with no brain MRI abnormalities only showed visual deficits, suggesting an I-ON. These two cases also showed normal CSF and were diagnosed with suspected optic neuritis based on the clinical signs and MRI features suggestive of optic nerve disease.

In contrast to this study in which all dogs had some imaging abnormality of the optic nerve, in a previous study (9) only a minority of the cases diagnosed with optic neuritis had MRI findings suggestive of optic nerve abnormality and 53.8% showed concurrent brain changes. In another recent study (2), 35% of dogs with confirmed I-ON had imaging lesions confined to the optic nerves however 83 % had abnormal imaging studies (not limited to the optic nerves) when diagnosed with optic neuritis as an extension of MUE. The high proportion of cases with concurrent brain and optic nerve abnormality in our study and previous studies (2, 9) could reflect the fact that dogs with optic neuritis and no other neurological deficits may present to veterinary ophthalmologists and not necessarily undergo an MRI study routinely. Alternatively, this could indicate that the isolated form of optic neuritis is less prevalent than the MUE-associated ON.

From the cases with suspected MUE-associated ON (cases that showed brain MRI changes, neurological signs suggestive multifocal brain disease and/or positive response to treatment), 46% (7/15) had a CSF result suggestive of inflammatory CNS disease and the remaining 54% (8/15) CSF results were normal, of which two cases had been treated with glucocorticoids before the sample was obtained. It has been reported that there is marked variability within the CSF results with some GME cases, with an estimated 10% showing normal protein concentration and normal leucocyte count (3). Additionally, prior treatment with glucocorticoids can reduce the likelihood of obtaining a positive CSF analysis (3). Our percentage of negative CSF results is higher than previously reported (3); this is likely to be due to the small sample in this study and not representative of the whole MUE population. Interestingly, both cases diagnosed with I-ON had a negative CSF sample result, which could be indicative of a more focal inflammation, however larger case numbers are needed to further analyse this finding.

There are several limitations in this study, mainly relating to its retrospective nature. The difference in sequences, orientation and protocols depending on the attending radiologist made some studies difficult to compare, although a core set of sequences were available in most cases for comparison. The radiologist reviewing the cases was blinded to the final diagnosis and any previous image interpretation and case data, however was aware of the inclusion criteria which could add some bias in the study. Three cases lacked a CSF sample and MUE was diagnosed based on the clinical signs, MRI findings and/or positive response to immunomodulatory treatment. Not all cases with a diagnosis of MUE had a positive CSF, since a negative CSF could be due to pre-treatment with glucocorticoids or be a normal finding as previously reported in some MUE cases; this precluded CSF being a gold standard against which to test the MRI findings. The use of menace response as a visual test for dogs can be non-objective in some cases, and this test was used in the majority of the cases to evaluate the vision. The use of a low field MRI scanner could be a limitation and some subtle lesions could have been missed because of the lower resolution. Not all of the previously reported infectious agents causing optic neuritis were tested for, however all of the dogs improved or were stable after immunosuppressive treatment, which makes an infectious etiology unlikely. Histopathology was not available for any of the cases and all the diagnosis were made based on clinical features and were therefore presumptive diagnosis. Finally, the small number of cases in our sample could be non-representative for the full spectrum of MRI findings in dogs with optic neuritis.

In conclusion, there were multiple subtle MRI abnormalities observed in the dogs included in this study that can indicate optic nerve pathology. Most of the abnormalities such as chiasmal and optic disc swelling could be appreciated in the sequences used routinely for the assessment of the brain but extra sequences focused on the optic nerves (such as a STIR or T1W post contrast dorsal oblique) can provide very useful additional information as both optic nerves can be directly compared allowing identification of subtle unilateral abnormality. Contrast enhancement was the most common finding in these dogs, therefore contrast is indicated in cases with suspicion of optic nerve involvement. Absence of CSF surrounding the optic nerve causing a lack of differentiation of the normal nerve sheath complex structures was observed in this study and could prove to be a useful additional feature to identify optic neuritis.

All of the sequences analyzed in this study (Table 2) showed some but not all of the features affecting the optic nerves or optic chiasm; and therefore the authors propose to maximize the chance of identifying MRI evidence of optic neuritis, that multiple sequences and planes are obtained. This study was not designed to evaluate which sequence is the most sensitive to optic nerve pathology, however since different patients demonstrated imaging features on differing sequences, the authors recommend that all cases having imaging evaluation of optic neuritis should have a pre and post contrast T1W sequence that allows comparison between optic nerves and/or a STIR dorsal oblique in addition to a standard brain protocol.

## Data Availability Statement

The original contributions presented in the study are included in the article/supplementary materials, further inquiries can be directed to the corresponding author/s.

## Ethics Statement

Ethical review and approval was not required for the animal study because it is a retrospective study. Written informed consent for participation was not obtained from the owners because a written informed consent to retain their animal's clinical information for use in future clinical studies was signed on hospital admission.

## Author's Note

Preliminary results from this study were presented in abstract form at the EAVDI—BID Pre BSAVA Meeting, April 2018.

## Author Contributions

AC and GC designed the study. LM acquired the data. LM and AC reviewed the studies, analyzed and interpreted the data and drafted the article. LM, AC, and GC read and approved the final manuscript. All authors contributed to the article and approved the submitted version.

## Conflict of Interest

The authors declare that the research was conducted in the absence of any commercial or financial relationships that could be construed as a potential conflict of interest.
